# HIV integrase and the swan song of the CD4 T cells?

**DOI:** 10.1186/1742-4690-10-149

**Published:** 2013-12-09

**Authors:** Jérôme Estaquier, John Zaunders, Mireille Laforge

**Affiliations:** 1 CNRS FRE 3235, Université Paris Descartes, Paris, France; 2 Université Laval, Centre de Recherche en Infectiologie, Québec, Canada; 3 St Vincent's Centre for Applied Medical Research, St Vincent's Hospital, Darlinghurst, NSW, Australia

**Keywords:** HIV, Apoptosis, DNA, p53, DNA-PK

## Abstract

T cell apoptosis represents one pathophysiological mechanism associated with AIDS. Herein, we discuss the recent report published by A. Cooper et al. in Nature (June 2013) regarding HIV viral DNA integration-mediated apoptosis.

## Background

Over twenty years ago it was hypothesized that CD4+ T lymphocyte depletion during HIV infection resulted from apoptosis [1,2], and since then numerous research teams have strived to identify the associated cellular and molecular mechanisms. As early as 1991, studies by Drs. D. Richman [3] and A. Hovanessian [4] showed that the virus induces apoptosis in lymphoblastoid T cell lines or mitogen-activated primary CD4+ T cells in vitro. This programmed cell death is independent of caspase activation [5-10], the main effector proteases involved in apoptosis [11].

## Main text

A paper recently published by A. Cooper *et al.* in Nature [12] suggests that integration of viral DNA is responsible for this apoptosis via activation of p53 by DNA-dependent protein kinase (DNA-PK), a protein kinase participating in DNA damage response. The authors show that apoptosis is only displayed by cells that do not express p24 antigen. An analysis of *in vitro* stimulated cells obtained from three HIV-infected individuals not receiving therapy suggested that the cells not expressing p24 antigen died in a proportion of 70 to >90%, but only 10 – 25% of these cells contained HIV DNA by qPCR. Furthermore, p24+ cells – representing 0.1-0.5% of CD4 T cells – died at a rate of 30–70%. This result implies that overall more than 90% of the CD4 T lymphocytes from HIV infected individuals underwent apoptosis in vitro, a proportion that seems to be incompatible with the in vivo status of these HIV-infected patients having CD4 cell counts up to 600 per mm^3^. The study also shows that blocking viral integration with raltegravir, an inhibitor of HIV integrase, decreased the occurrence of cell death not only in T/B lymphoblastoid cell line CEMX174, but also in primary CD4 T cells activated with PHA/IL-2. Likewise, a virus bearing a mutated integrase (D64V) caused less apoptosis. The authors propose that viral integration was responsible for cell death. Thus, lymphocytes would die before the virus gets a chance to replicate. Considering this, one has to wonder what advantage a pathogenic agent may gain from such a mechanism. Previous work by Dr. F. Bushman [13] had shown that it may be the accumulation of viral DNA and not its integration that could induce this apoptosis during activation of CD4+ T lymphocytes. This accumulation of viral DNA has also been proposed to induce the death of T cells in a human tonsil model [14] – described as early as the 1990s by Margolis’ group as supporting viral replication [15]. This process is accompanied by chronic inflammatory response that can be associated with caspase-1 activation, a caspase involved in pyroptotic cell death [16].

Cooper *et al.* furthermore propose that a phosphorylation of proteins p53 and H2AX accompanies this process via DNA-PK activation. Pharmacological inhibition of DNA-PK activation not only prevents phosphorylation of these two molecules, but cell death as well. The role of the DNA-PK pathway is largely studied in the context of double-strand break repair through non-homologous end joining (NHEJ). In 1999, Daniel and colleagues [17] reported that DNA-PK activity increases as a consequence of retroviral integration. The authors also showed that an HIV-1–based virus vector induced death in scid pre-B cell lines This death was proposed to be due to a defect in DNA-PK in these cell lines, resulting in a lack of DNA repair needed to complete the retroviral integration. Several groups subsequently showed that DNA damage sensors ATM, ATR, DNA-PK, and PARP-1 were, however, not required for efficient HIV-1 integration [18,19], and a protective role of DNA-PK was only observed against death induced by high levels of retrovirus integration. Thus, DNA-PK may exert a protective effect on the infected cells, a claim exactly opposite to that of Cooper and colleagues. Moreover, it has been reported that the activity of HIV-1 integrase stimulates an ATM-dependent DNA damage response, and that a deficiency of this kinase sensitizes cells to retrovirus-induced cell death [20]. Paradoxically, the inhibitor used in that study was KU55933, which was the same molecule utilized by Cooper and colleagues to show that ATM inhibition does not relieve cell death upon HIV infection. A possible explanation of such controversial results should be the difference of cells used in these studies, cell lines versus primary T cells.

Lastly, the authors show that inhibiting p53 activation with a pharmacological agent, pifithrin, also blocks CD4 T cell apoptosis. However, the nature of the cells expressing p53 and DNA-PK was not assessed by the authors, although implicitly they suggested p24- cells. On the contrary, several groups, including ours, have previously shown that phosphorylation of p53 and expression of target genes only occurred in cells replicating the virus (p24+) [21-23]. We have also recently shown that silencing p53 with interfering RNA reduces apoptosis [23] and increases viral replication in primary CD4 T cells. Therefore, we favour the hypothesis that p53 activation constitutes a stress-sensing mechanism, allowing auto-elimination of infected cells, and thus a host altruistic defence mechanism limiting viral dissemination. This programmed cell death is associated with lysosomal destabilization [10,23], which requires viral replication, since bystander cells – exposed to the virus, but not infected – do not display lysosomal destabilization.

Although increased activation of CD4 T cells during HIV-1 infection promotes viral production, the fact remains that the proportion of productively infected CD4 T cells in lymphoid tissue is very low, 0.25 - 1% or less of the cells that contain HIV DNA, which in turn represent about 10 - 20% of all CD4 T cells (reviewed in [24]). Much of this HIV DNA, at least in circulating memory CD4 T cells during untreated HIV infection, is in a labile unintegrated linear form or in episomal 2-LTR circles, [25]. After extended antiretroviral treatment of HIV-infected subjects, the cells containing integrated HIV DNA are long-lived [25,26]. The analysis of related simian immunodeficiency viruses (SIV), comparing those that are pathogenic for their hosts, such SIVmac239 or SIVmac251, to the non-pathogenic SIVagm, SIVsm or SIVmnd, has shown that, despite a sustained viral replication in both pathogenic and non-pathogenic infections, only pathogenic models display an exacerbated apoptosis of CD4 T lymphocytes [27-30], beginning during primary infection [31-33]. Studies have shown that non-infected cells die mostly by apoptosis [34,35]. The level of apoptosis is a predictor of the rate of progression to AIDS and correlates with the innate immune response [36,37]. Furthermore, other work has shown in patients said to be discordant with respect to their immunovirological status - i.e. individuals for whom antiviral therapy is efficient, but in whom CD4 depletion continues – that there is an abnormal level of CD4+ T lymphocyte apoptosis [38]. Taken all together, it seems unlikely that integration of HIV DNA *per se* is responsible for the apoptosis observed in lymphoid tissues in vivo and clinically relevant CD4 depletion.

Considering the various non-human primate (NHP) models, it is noteworthy that the immune response of CD4 T lymphocytes of AGM monkeys or sooty mangabeys is extremely limited [29,39,40] and that their weakly activated central memory cells contain most of the viral DNA [41]. Several studies have indeed shown that chronic activation of the immune system may induce an activation-induced cell death (AICD)-type apoptosis via death receptors, particularly Fas and its ligand [32,42-48], with the latter being more weakly expressed in non-pathogenic infection models [49]. Furthermore, a role has been proposed for Trail and its death receptors, TRAIL-R1/R2, via type-1 interferons [50], although this work remains controversial [46,49,51,52]. Moreover, Trail has little influence on T lymphocyte homeostasis, as opposed to Fas, or the major part played by Bim, a pro-apoptotic member of the Bcl-2 family [53-55]. A role for Bim has been described, first in NHPs infected by SIV [46], but also in HIV patients [56]. Therefore, increased apoptosis could be due to activation, rather than infection of cells by HIV-1. However, addition of cyclosporine A, an inhibitor of T cell activation, to antiretroviral therapy (ART) does not provide apparent virologic or immunologic benefit [57].

Furthermore, the absence of co-signals by APCs [58] and the production of immunosuppressive cytokines, such as interleukin-10 or TGF-β, may trigger T cell apoptosis [43,59,60] involving Bim. It is noteworthy that adding exogenous factors, such as interleukins-2, -12 or −15, can prevent apoptosis of CD4 T lymphocytes *ex vivo*[44,61,62], via induction of anti-apoptotic cellular factors such as Bcl-2 or Bcl-x, antagonists of Bim. However, immunotherapy based on IL-2 did not yield any benefit during the chronic phase of either HIV or SIV-infections [63,64].

IL-7 is the most important anti-apoptotic exogenous signal for T cell survival *in vivo*. We have shown directly that highly activated CD8 T cells in subjects during primary HIV-1 infection have reduced IL-7 receptor and reduced Bcl-2 and rapidly undergo apoptosis spontaneously *in vitro*, unless cultured with the common gamma-chain cytokines IL-2 or IL-15 [65]. However, IL-7 signalling may additionally enhance integration of HIV-1 into the genome of target CD4 T cells [66] as well as re-activation of productive infection from latently infected cells [67]. Furthermore, its impact on CD4 T death seems to depend on the duration of exposure to this cytokine or on production of accessory cytokines. It indeed displays no discernible effect on purified CD4 T lymphocytes [44], whereas added to PBMCs it prevents apoptosis after 4 days of culture [68].

Increased IL-7 levels in progressive HIV-1 infection may also increase the expression of the coreceptor CXCR4, resulting in a greater risk of emergence of an X4-using viral strain [69], and in turn IL-7 may prove to be more deleterious and pro-apoptogenic with these X4 viral strains, as it causes an increase of Fas [70-72]. Not only does the interaction between the viral envelope and the CD4 molecule prime the cells to undergo apoptosis [73-77], but interaction with viral coreceptors CCR5 and CXCR4 may also induce apoptosis and enhance Fas-mediated cell death independently of immune activation [32,78-80]. Given this possible role of co-receptors in initiating apoptosis, does Maraviroc, a CCR5 inhibitor, have an enhanced role *in vivo* in inhibiting CD4 T cell death?

Considering NHP models, a simple question must be asked –WHY do these viruses, albeit “cytolytic” after *in vitro* stimulation, not cause in their hosts a very rapid depletion of CD4 T lymphocytes, in all models of infection? In fact, based on the suggestion by Cooper *et al.* that every cell integrating HIV DNA automatically dies, non-pathogenic SIV infection in NHP should not be possible. Moreover, what would be the possible advantage for the virus to activate and deplete the immune system if this leads to a very rapid death of its host? It should be remembered that HIV-1 infection is a recent zoonosis, to which HIV-1 has presumably not yet completely adapted. Clearly the activation of the immune system that results from HIV-1 infection may increase target cells for productive infection in the short term, but may also relatively rapidly lead to the demise of the host and presumably reduce overall the chance of transmission (notwithstanding the size of the HIV-1 pandemic). Conversely, does a non-pathogenic SIV in its natural host intentionally limit lymphocyte activation more than the strong activation of a pathogenic strain of SIV or HIV? Is the virus trying to minimize the CD4 response, to render it relatively anergic, in order to facilitate its own dissemination? Likewise, does the localization of the virus into sites where the immune response is tightly controlled, such as the intestine, represent an evasion strategy, a way to hide in sanctuaries characterized by weak activation? This could account for viral persistence in HIV-infected patients in spite of several years of highly-active therapy. Therefore, like Orpheus do non-pathogenic viruses render the cells resistant to the song of death.

## Conclusions

In conclusion, this new study from Cooper *et al.* proposes an apoptosis molecular mechanism linked to viral integration associated with the activation of p53 and DNA-PK. However, this concept is difficult to reconcile with known *in vivo* events, and, furthermore, an eventual therapeutic strategy aimed at blocking p53 or DNA-PK could be highly risky, by promoting either viral replication or cancer. Due to the complexity of the biochemical apoptotic pathways described leading to CD4 cell death, inhibiting this process *in vivo* presents a real challenge.

## Competing interests

The authors declare that they have no competing interests.

## Authors’ contributions

EJ, JZ, and LM wrote the manuscript. All authors read and approved the final manuscript.
